# Temporal dynamic characteristics of human monkeypox epidemic in 2022 around the world under the COVID-19 pandemic background

**DOI:** 10.3389/fpubh.2023.1120470

**Published:** 2023-01-26

**Authors:** Yanxiang Cao, Meijia Li, Naem Haihambo, Xinni Wang, Xixi Zhao, Bin Wang, Meirong Sun, Mingrou Guo, Chuanliang Han

**Affiliations:** ^1^The National Clinical Research Center for Mental Disorders and Beijing Key Laboratory of Mental Disorders, Beijing Anding Hospital, Capital Medical University, Beijing, China; ^2^Advanced Innovation Center for Human Brain Protection, Capital Medical University, Beijing, China; ^3^Faculty of Psychology and Center for Neuroscience, Vrije Universiteit Brussel, Brussels, Belgium; ^4^Faculty of Psychology, Beijing Normal University, Beijing, China; ^5^School of Psychology, Beijing Sport University, Beijing, China; ^6^Shenzhen Key Laboratory of Neuropsychiatric Modulation and Collaborative Innovation Center for Brain Science, Guangdong Provincial Key Laboratory of Brain Connectome and Behavior, CAS Center for Excellence in Brain Science and Intelligence Technology, Brain Cognition and Brain Disease Institute, Shenzhen Institute of Advanced Technology, Chinese Academy of Sciences, Shenzhen–Hong Kong Institute of Brain Science, Shenzhen Fundamental Research Institutions, Shenzhen, China

**Keywords:** monkeypox, sigmoid function, epidemics, public health, COVID-19

## Abstract

**Background:**

The reemergence of the monkeypox epidemic has aroused great concern internationally. Concurrently, the COVID-19 epidemic is still ongoing. It is essential to understand the temporal dynamics of the monkeypox epidemic in 2022 and its relationship with the dynamics of the COVID-19 epidemic. In this study, we aimed to explore the temporal dynamic characteristics of the human monkeypox epidemic in 2022 and its relationship with those of the COVID-19 epidemic.

**Methods:**

We used publicly available data of cumulative monkeypox cases and COVID-19 in 2022 and COVID-19 at the beginning of 2020 for model validation and further analyses. The time series data were fitted with a descriptive model using the sigmoid function. Two important indices (logistic growth rate and semi-saturation period) could be obtained from the model to evaluate the temporal characteristics of the epidemic.

**Results:**

As for the monkeypox epidemic, the growth rate of infection and semi-saturation period showed a negative correlation (*r* = 0.47, *p* = 0.034). The growth rate also showed a significant relationship with the locations of the country in which it occurs [latitude (*r* = –0.45, *p* = 0.038)]. The development of the monkeypox epidemic did not show significant correlation compared with the that of COVID-19 in 2020 and 2022. When comparing the COVID-19 epidemic with that of monkeypox, a significantly longer semi-saturation period was observed for monkeypox, while a significant larger growth rate was found in COVID-19 in 2020.

**Conclusions:**

This novel study investigates the temporal dynamics of the human monkeypox epidemic and its relationship with the ongoing COVID-19 epidemic, which could provide more appropriate guidance for local governments to plan and implement further fit-for-purpose epidemic prevention policies.

## Introduction

Monkeypox, a member of the genus *Orthopoxvirus*, was discovered in 1958 when a pox-like disease occurred in monkeys (1, 2). Subsequently, in 1970 the first animal-to-human transmission was discovered after monkeypox was identified in a 9-month-old boy in the Democratic Republic of Congo (3), demonstrating that monkeypox is a severe viral zoonotic disease (4). Since then, several outbreaks of monkeypox have been reported in many countries, which occur mostly in central and western African countries (5–10), close to tropical rain forests. Clinically, some studies suggest that the current monkeypox outbreak presents lesions on genital and perianal areas (11–14). Although less severe, the monkeypox virus presents with a pustular rash similar to that seen in presentations of smallpox (10, 15–17), with the exception that monkeypox causes swelling in the lymph nodes (14).

The monkeypox epidemic demonstrates a tendency toward becoming a worldwide pandemic, similar to the initial stages of COVID-19. Previous travel-associated occurrences outside of Africa have shown limited secondary spread (18), and therefore human-to-human transmission has been deemed inefficient. However, demonstrating the recrudescence property of infectious diseases (19–22) the monkeypox epidemic recently reemerged in Nigeria since early May 2022 and spread quickly to more than 50 countries across five regions, such as the United Kingdom and many other countries around the world that are not endemic to this virus (3, 23–25). Considering the trajectory of the current rising COVID-19 pandemic, the precise temporal dynamic characteristics of the monkeypox epidemic should be investigated. Notably, previous studies have also used similar dynamic characteristics, semi-saturation period of the epidemic was widely used (26–28), which is similar to the concept of the inflection point that people are concerned about. This index is intuitive to mark the important turning point of the epidemic.

As the monkeypox epidemic spreads, the COVID-19 pandemic is still ongoing around the world (29). COVID-19 cases were first reported in December 2019 (30–33) and then confirmed human-to-human transmission (31, 34–44). In most countries worldwide, multiple waves of the epidemic have been observed (45–54). To control the global pandemic, countries implemented prevention policies. Studies have suggested that COVID-19 prevention measures could also have a positive effect on other infectious diseases (55–59). Additionally, both COVID-19 and monkeypox are zoonotic diseases (60), which means that they can be transmitted between species from animals to humans. However, it is still unclear whether similar approaches may also be employed to combat the monkeypox outbreak. To determine this, we need to explore the underlying growth mechanism and figure out whether the development of monkeypox and COVID-19 epidemic have similarities.

Hence, in this study, we aimed to explore the temporal dynamic characteristics of the human monkeypox epidemic in 2022 and its relationship with those of the COVID-19 epidemic. A simple and effective descriptive model was established to fit the time series data of the cumulative number of infected cases with monkeypox and COVID-19. We further summarized the basic properties of the model parameters [growth rate and semi-saturation period (the inflection point of the sigmoid curve)] and compared them between the monkeypox and COVID-19 epidemic.

## Methods

### Data and sources

Available time series data for the daily reported and confirmed cases of monkeypox and COVID-19 in 21 countries (Austria, Belgium, Brazil, Canada, Chile, Denmark, France, Germany, Ireland, Israel, Italy, Mexico, Netherlands, Nigeria, Peru, Portugal, Spain, Sweden, Switzerland, the United Kingdom, and the United States) from 6th May to 12th August 2022 were extracted from situation reports on the official website of the WHO. The main reason for choosing these countries is that their monkeypox epidemic is relatively serious (the cumulative number of infected cases is larger than 50). In order to compare the temporal dynamics of the initial stage, time series data for the daily reported and confirmed cases of COVID-19 in 21 countries in 2020 were also extracted from official website of WHO with the same duration. The dataset is open to the public worldwide. The latitude and longitude of each country (represented by geodetic centroids) were obtained from an open-source website (https://github.com/gavinr/world-countries-centroids). The Gross Domestic Product (GDP) and the World Development Indicators (WDI) data of the 21 countries in 2021 were obtained from the world band websites (https://databank.worldbank.org), which represent the most commonly used measure for the size of an economy and comparable cross-country data on economic development.

### Ethical considerations

For this study, the data we use are all publicly available. Our study did not involve any intervention on human participants. This study was approved by the Ethics Committee of Beijing Sport University (2022142H), China.

### Data preparation process

After getting the daily data of monkeypox and COVID-19 cases, we first transformed the daily data into cumulative data from the beginning of the date. During this process, we also checked whether there were outliers in the dataset (3-sigma principle). All the data of infected cases did not indicate outliers. Modeling was done after these steps.

### Descriptive model of epidemics

We used the logistic model (sigmoid) to capture the temporal dynamics of the monkeypox and COVID-19 epidemic in 21 countries (Eq. 1).


(1) $$\begin{array}{l} N\left(t\right)=\frac{A}{{1+e}^{-k\;\left(t-t_{0}\right)}} \end{array}$$

Where, *N* (*t*) is the general form of the cumulative number of infected patients at time *t, A* denotes the maximum number of infections, *k* is the logistic growth rate, and *t*_0_is the semi-saturation period (SSP), that is, the mathematically defined inflection point of the sigmoid curve.

We processed the data and modeled them with custom scripts on MATLAB (the MathWorks). We adopted the non-linear least squares (NLS) algorithm for data fitting and parameter estimation, which have been widely used in the previous studies in describing the epidemical dynamics (27, 28). The goodness of fit was defined as formula (2).


(2) $$\text{Goodness of fit}=1-\frac{\sum_{1}^{n}\;{\left(R_{data}\left(i\right)-R_{model}\left(i\right)\right)}^{2}}{\sum_{1}^{n}{\left(R_{data}\left(i\right)-\frac{\sum_{1}^{n}R_{data}\left(i\right)}{n}\right)}^{2}}$$

### Correlation analysis

We used the Pearson correlation to measure the relationship between logistic growth rate (*k*) and semi-saturation period (*t*_0_) in monkeypox epidemic. The Pearson correlation was also used in the correlation analysis between model parameters (*k, t*_0_) and the location of the country (longitude and latitude). The Pearson correlation was further used in the correlation between model parameters in monkeypox epidemic and COVID-19 epidemic.

### Statistical analysis

We used one-way ANOVA test to compare the model parameters (*k, t*_0_) between monkeypox epidemic and COVID-19 epidemic (ongoing and its initial stage in 2020), and the model parameters (*k, t*_0_) in different climate types.

## Results

### Temporal dynamics of the human monkeypox epidemic

**Figure 1:**
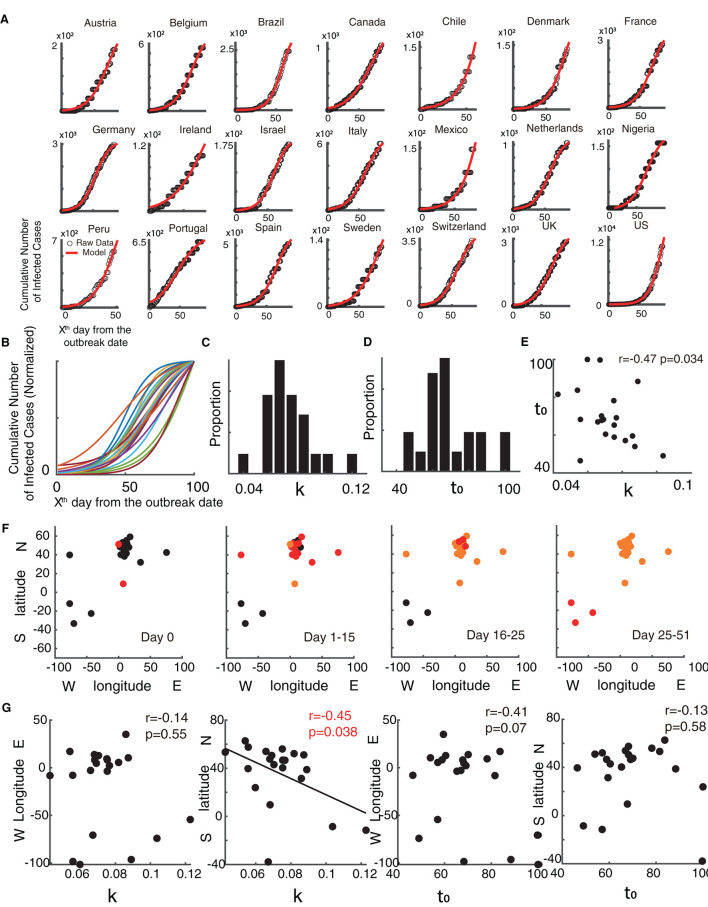
Temporal dynamics of the monkeypox epidemic. **(A)** Raw data of the cumulative infected cases of monkeypox is shown in black dots and the fitted curve of the model is shown in red. **(B)** Normalized fitted curves of model for 21 countries. **(C)** Distribution of growth rate (k). **(D)** Distribution of semi-saturation period (t_0_). **(E)** Relationship between growth rate (k) and semi-saturation period (t_0_). **(F)** Spatial location of 21 countries and the development of the spread in four stages (Day 0, Day 1–15, Day 16–25, Day 26–51). The red dots are new outbreak countries in the current stage, the orange dots show countries where the virus is endemic, and the black dots indicate countries that have not experienced the epidemic. **(G)** Relationship between the spatial location (geodetic centroid) with the model parameters [growth rate (k) and semi-saturation period (t_0_)].

Time series data of the monkeypox from 21 countries could be explained well (Figure 1A, Goodness of fit > 0.95) using the descriptive model (see Methods). After normalizing the fitted curve (Figure 1B), we found that the fitted curves of the monkeypox epidemic are variable among countries based on growth rate and semi-saturation period (Figures 1C, D). The growth rate (k) and semi-saturation period (t_0_) of the monkeypox epidemic showed a negative correlation, indicating that the higher growth rate is correlated with lower semi-saturation period (Figure 1E, *r* = 0.47, *p* = 0.034). Panel F of Figure 1 showed the spatial extension of the monkeypox epidemic in the initial stage.

We then explored whether these temporal properties have any relationship with the longitude and latitude coordinates of the country (Figure 1G).

Results found that the growth rate (k) of the monkeypox epidemic showed significant relationships with the locations of the country latitude (*r* = –0.45, *p* = 0.038), but not for longitude (*r* = −0.14, *p* = 0.55), showing that the higher growth rate is correlated with lower latitude. Geologically, these regions are close to the equator (0-degree latitude) and the prime meridian (0-degree longitude). However, no significant correlation was found when comparing the semi-saturation period (t_0_) and spatial locations.

### Temporal dynamics of the contemporaneous COVID-19 epidemic

**Figure 2:**
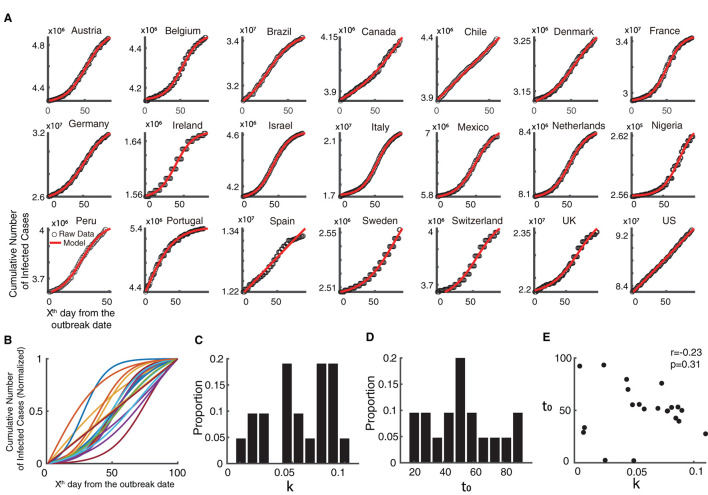
Temporal dynamics of the ongoing COVID-19 epidemic. **(A)** Raw data of the cumulative infected cases of COVID-19 is shown in black dots and the fitted curve of the model is shown in red, in the same time interval as the monkeypox epidemic. **(B)** Normalized fitted curves of model for 21 countries. **(C)** Distribution of growth rate (k). **(D)** Distribution of semi-saturation period (t_0_). **(E)** Relationship between growth rate (k) and semi-saturation period (t_0_).

Meanwhile, time series data of the COVID-19 from 21 countries at the same time window (from 6th May to 12th August 2022) could be explained well (Figure 2A, Goodness of fit > 0.95) using the descriptive model (see Methods). Similar to that of the monkeypox epidemic, the normalized fitting curves of the monkeypox epidemic are variable among countries based on growth rate and semi-saturation period (Figures 2B–D). The growth rate (k) and semi-saturation period (t_0_) of the COVID-19 epidemic showed no significant correlation (Figure 2E, *r* = 0.23, *p* = 0.31). We also explored the relationship between temporal properties of the COVID-19 epidemic and the longitude and latitude coordinates of the country, and we did not find any significance.

**Figure 3:**
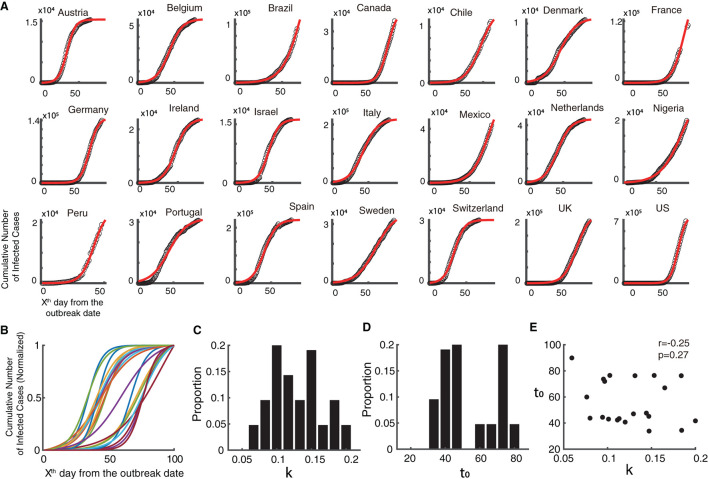
Temporal dynamics of the COVID-19 epidemic in 2020. **(A)** Raw data of the cumulative infected cases of COVID-19 is shown in black dots and the fitted curve of the model is shown in red, in 2020 (initial stage of the epidemic). **(B)** Normalized fitted curves of model for 21 countries. **(C)** Distribution of growth rate (k). **(D)** Distribution of semi-saturation period (t_0_). **(E)** Relationship between growth rate (k) and semi-saturation period (t_0_).

At present, COVID-19 and monkeypox share the same period. However, in the development stage, the former may have been well known and implemented effective prevention strategies, while the latter is still spreading in ways not previously seen. Hence, we further used time series data of COVID-19 from the same 21 countries in its initial stage of 2020, and found that it could be explained well (Figure 3A, Goodness of fit > 0.95) using the descriptive model. The normalized fitting curves of the COVID-19 epidemic vary among countries based on growth rate and semi-saturation period (Figures 3B–D). The growth rate (k) and semi-saturation period (t_0_) of the COVID-19 epidemic showed no significant correlation (Figure 3E, *r* = 0.25, *p* = 0.27).

### Comparison between the temporal properties of monkeypox and COVID-19 epidemics

**Figure 4:**
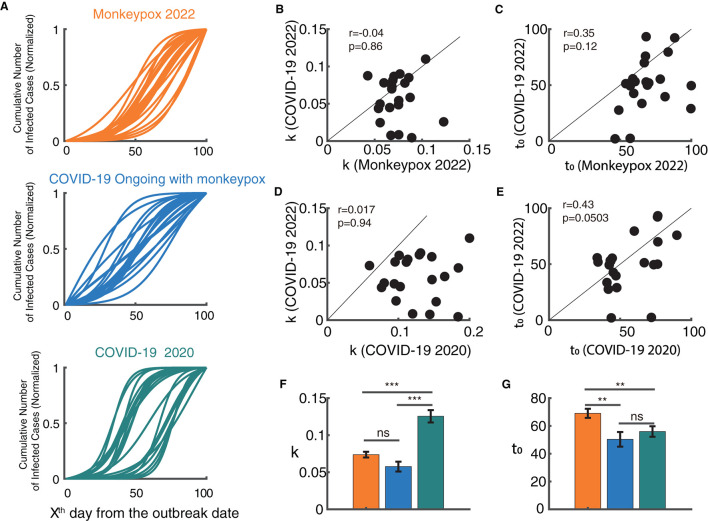
Relationship between the model parameters of monkeypox and the COVID-19 epidemic. **(A)** Normalized fitted curves of model for 21 countries in monkeypox 2022 (blue), COVID-19 2022 (orange) and COVID-19 2020 (green). **(B, C)** Relationship between growth rate (k) and semi-saturation period (t_0_) between monkeypox and COVID-19 in 2022. **(D, E)** Relationship between growth rate (k) and semi-saturation period (t_0_) between COVID-19 in 2020 and COVID-19 in 2022. **(F, G)** Comparison of the values of growth rate (k) and semi-saturation period (t_0_) between monkeypox and COVID-19 in both stages.

After capturing the temporal properties of the monkeypox and COVID-19 epidemics, we then explored their similarities and differences (Figure 4). It is obvious that the COVID-19 epidemic and the monkeypox outbreak are evolving in quite distinct ways (Figure 4A). The temporal dynamic characteristics (k and t_0_) of the monkeypox outbreak did not show any significant correlation with the ongoing COVID-19 in 2022 (Figures 4B, C). Similarly, the temporal dynamic characteristics of COVID-19 in 2020 also did not show any significant correlation with the ongoing COVID-19 in 2022 (Figures 4D, E). However, the growth rate of the monkeypox epidemic was significantly shorter than that of the COVID-19 epidemic in 2020 (*p* < 0.001), but showed no significant difference from the ongoing COVID-19 epidemic (p>0.05) (Figure 4F). Additionally, the semi-saturation period (t_0_) of the monkeypox epidemic was significantly longer than that of the ongoing COVID-19 epidemic (*p* < 0.01) and the COVID-19 epidemic in 2020 (*p* < 0.01) (Figure 4G). According to these findings, the growth rate of the monkeypox may have been relatively comparable COVID-19 during the same time, but it may have taken longer for the monkeypox outbreak to reach the semi-saturation point.

## Discussion

### Principal findings

In the present study, we used an effective descriptive model to successfully explain temporal dynamics using available public data on monkeypox and COVID-19 epidemics. We demonstrated that this model is able to capture the global macroscopic dynamics of epidemics. We also explored the basic properties of their development and found some common characteristics and notable differences. With these findings, we could better provide the government with recommendations on the optimal intervention timing, thereby helping to design fit-for-purpose policies. In the monkeypox epidemic, we also discovered the association between the temporal dynamics and the locations of the countries (longitude and latitude), and found the growth rate of the monkeypox epidemic also showed a significant relationship with specific longitudinal coordinates, although not for the semi-saturation period. Regarding latitude, temperatures tend to be warmer in the Equator and cooler near the Poles, which may account for the different growth rates. Evidence has also demonstrated that greater incidence is recorded from regions with higher mean temperatures and less yearly precipitation. More specifically, we classified these countries into five climate types (Figure S1D). We did not find significant differences among them, but did find a tendency that the countries in tropical climate have a higher growth rate and the countries in temperate continental and subtropical climate have a longer semi-saturation period, which could point to a difference. Taken together, these results suggest that the temporal dynamics of the monkeypox epidemic might be related to the geological environment (61, 62).

The comparison between the monkeypox outbreak and the COVID-19 epidemic revealed that the temporal dynamic characteristics do not correlate in these two outbreaks, which indicates that the temporal dynamics of the COVID-19 were not followed by the monkeypox outbreak. It implies two different temporal mechanisms for the diseases to propagate. Additionally, combining the two COVID-19 outbreaks in different time stages, there was no discernible association between the COVID-19 epidemics in 2020 and 2022, suggesting that after 2 years of mutation, the diseases own temporal features are also altering. By comparing the temporal properties of the three periods of epidemics (2022 monkeypox, 2022 COVID-19 and 2020 COVID-19) (Figures 4F, G), we found the growth rate of 2020 COVID-19 epidemic is the largest, and the semi-saturate period of 2022 monkeypox is the largest. Based on the previous theory (27), the growth rate may reflect the natural property of the virus, and the semi-saturation period reflects the effectiveness of the government policy, but a more detailed data is needed for analysis in the future.

### Comparison with prior work

To our knowledge, this is the first study to systematically investigate the temporal dynamics of the new monkeypox epidemic in 2022 around the world. Previous studies focused more on the monkeypox epidemic before 2020 and only in a few countries in Africa (5–10). While the virus is endemic to western and central Africa, the current 2022 outbreak has spread to non-endemic areas in Europe, America, and Asia. It has increasingly become an important public health event that cannot be ignored. Taking into account the global scale at which COVID-19 has spread, and the time overlap between the monkeypox and COVID-19, it is important to understand the temporal dynamics of monkeypox, and how they may be distinct from or comparable with COVID-19. Our study attempts to investigate these dynamics and fill this gap.

At a modeling level, mathematical models have seldom been used in understanding the temporal dynamics of the monkeypox outbreak, but there are some descriptive statistics (63). A previous study (64) used a complex dynamical system model with differential equations to simulate the transmission dynamics of monkeypox. However, while their study could be useful in proving the benefits of isolation in reducing disease transmission, the complex dynamical model was restricted to simulations and could not be fitted to real data. The model we used, however, could be combined with real data. All parameter estimations are grounded in real data, which has strong practical implications. Aside from that, this model has also been developed over time and has been used in many studies related to infectious diseases (27, 28, 65) with a sound theoretical basis.

### Limitations

One limitation of our study is that the data we used is the country-level data, which did not have a high spatial resolution that province-level (equivalent) data could have provided. In addition, we did not adopt the incidence regions but merely the longitude and latitude of the capital city, which may have obscured certain information. Future work should provide more data specificity at the province or city level to precisely describe the temporal dynamics and geological features. An additional limitation is that our model could not explain epidemics of more than one wave. If there are multiple waves in the future, we would revise the current model to better account for these fluctuations and to better explain the data. This study focused on the initial stage of the monkeypox outbreak dynamics, which is still ongoing. Once the epidemic had subsided or stabilized, a follow-up study could investigate the panoramic development of the monkeypox epidemic over time and how it could have differed from or been comparable to the COVID-19 pandemic. In the end, there are multiple factors that may have a profound impact on government decisions, such as economic factors (Figures S1A–C). The current study did not specifically focus on economic and financial factors; however, future studies should focus on these for a clearer picture.

## Conclusion

In summary, using a descriptive model, we captured the global macroscopic dynamics of monkeypox and COVID-19 epidemics, and compared their relationship with economic and climate factors. This analytic framework could provide the government with more appropriate recommendations on optimal intervention timing, thereby helping to design fit-for-purpose policies for new epidemics in the future.

## Data availability statement

The datasets presented in this study can be found in online repositories. The names of the repository/repositories and accession number (s) can be found in the article/Supplementary material.

## Author contributions

CH, ML, XZ, and YC conceived and designed the study. CH, ML, NH, BW, MS, and XW contributed to the literature search. CH and ML contributed to data collection, data analysis, and the interpretation of results. All authors contributed to the article and approved the submitted version.
